# Awareness, Treatment, and Control of Diabetes in Bangladesh: A Nationwide Population-Based Study

**DOI:** 10.1371/journal.pone.0118365

**Published:** 2015-02-18

**Authors:** Md. Shafiur Rahman, Shamima Akter, Sarah Krull Abe, Md. Rafiqul Islam, Md. Nazrul Islam Mondal, J. A. M. Shoquilur Rahman, Md. Mizanur Rahman

**Affiliations:** 1 Department of Public Health, First Capital University of Bangladesh, Chuadanga, Bangladesh; 2 Department of Epidemiology and Prevention, Center for Clinical Sciences, National Center for Global Health and Medicine, Tokyo, Japan; 3 Department of Global Health Policy, University of Tokyo, Tokyo, Japan; 4 Department of Population Science and Human Resource Development, University of Rajshahi, Rajshahi, Bangladesh; Stanford University, UNITED STATES

## Abstract

**Objectives:**

To examine awareness, treatment, and control of diabetes mellitus among the adult population in Bangladesh.

**Methods:**

The study used data from the 2011 nationally representative Bangladesh Demographic and Health Survey (BDHS). The BDHS sample is comprised of 7,786 adults aged 35 years or older. The primary outcome variables were fasting blood glucose, diagnosis, treatment, and control of diabetes. Multilevel logistic regression models were used to identify the risk factors for diabetes awareness.

**Results:**

Overall, age-standardized prevalence of diabetes was 9.2%. Among subjects with diabetes, 41.2% were aware of their condition, 36.9% were treated, and 14.2% controlled their condition. A significant inequality in diabetes management was found from poor to wealthy households: 18.2% to 63.2% (awareness), 15.8% to 56.6% (treatment), and 8.2% to 18.4% (control). Multilevel models suggested that participants who had a lower education and lower economic condition were less likely to be aware of their diabetes. Poor management was observed among non-educated, low-income groups, and those who lived in the northwestern region.

**Conclusions:**

Diabetes has become a national health concern in Bangladesh; however, treatment and control are quite low. Improving detection, awareness, and treatment strategies is urgently needed to prevent the growing burden associated with diabetes.

## Introduction

Diabetes mellitus is a major global health problem, affecting 382 million people, accounting for 5.3 million deaths in 2013 [1–3]. By 2035 the number of affected people is expected to increase to 592 million globally [1,4]. About 80% of adults with diabetes live in low- and middle-income countries [1]. Diabetes has become the seventh leading attributable risk factor for burden of disease in South Asian countries [5]. Previously diabetes was a disease of the affluent, but now it has become a major public health problem in low- and middle-income countries [6–8], particularly affecting South Asians [4]. The economic and disease burden associated with non-communicable diseases especially diabetes puts enormous pressure on fragile health systems in low-income countries [6,9–11]. In the South Asian region, Bangladesh has the second largest number of adults with diabetes (5.1 million adults, 6.31%) [1]. Therefore, understanding the extent to which households or populations are not being diagnosed, treated, and controlling their diabetes condition may reveal opportunities to reduce premature death, disability, and household economic shock.

Several studies, mainly from high- and middle-income countries, have shown that the rate of diabetes is increasing, but diagnosis, treatment, and control are quite low [12–18]. Even in the USA, more than one fourth of people aged 20 to 79 years with diabetes were unaware of their condition in 2008 [19]. Some studies from China reported that despite the high prevalence of diabetes, less than half the people were aware, and very few controlled their condition [14,16,20]. A recent multi-country study found that people in low-income countries and those with lower economic profile were less likely to receive a timely diagnosis and treatment for their non-communicable diseases (NCDs) [13]. However, the previous multi-country study was limited to few covariates and mainly included low-income countries in Africa [4]. Little is known about the extent of diabetes management in low-income settings such as Bangladesh, where diabetes has become a major health concern, prevalence is increasing, access to care is limited, and a significant proportion of households borrow money or sell household assets to cope with diabetes related treatment costs [1,5,21].

Although prevalence and risk factor assessment is not rare in Bangladesh [21–26], the research on diabetes management especially diagnosis, treatment, and control is nonexistent. This is the first attempt to estimate prevalence of awareness, treatment, and control of diabetes using nationally representative survey data. We additionally investigated the variation of diagnosis for diabetes using multilevel regression models with random intercept terms at household and community level.

## Methods

### Ethics statement

We obtained the data used in this study from MEASURE DHS Archive. The data were originally collected by the Macro, Calverton, USA. The authors are grateful to Measure DHS for providing permission to use the 2011 Bangladesh DHS data.

### Study population

Bangladesh is one of the most densely populated countries in the world (1,015 people per sq km), with a population of nearly 149.8 million in 2011 [27]. The country has seven administrative regions: south (Barishal), southeast (Chittagong), central (Dhaka), west (Khulna), mid-western corner (Rajshahi), northwest (Rangpur), and east (Sylhet). These regions display different geographic, demographic, environmental, culinary, and economic features [27–30]. The socio-economic status of the southern part of Bangladesh lags behind other regions. Residents in the northwestern part of Bangladesh suffer disproportionately more from poverty, malnutrition, and other socio-economic indicators [27,29,30].

### Study design

**Fig 1:**
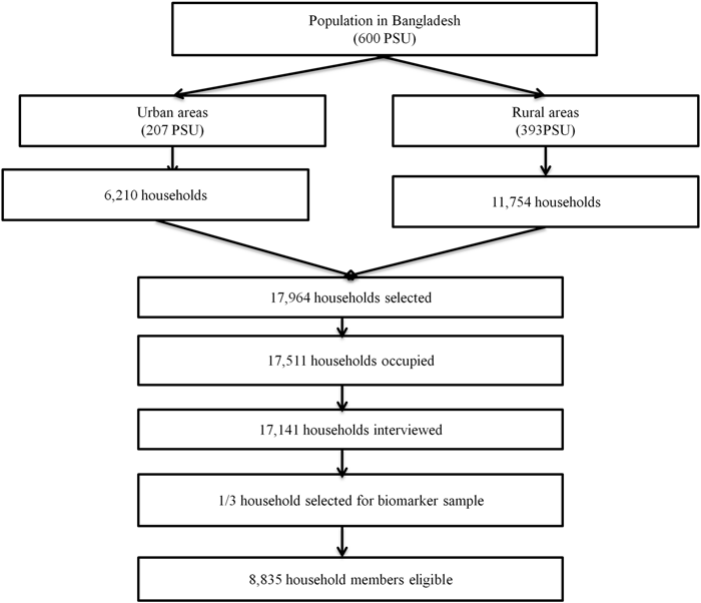
Sampling process.

This research used data available as of February 2013 from the Bangladesh Demographic and Health Survey (BDHS). The survey was conducted between July and December 2011 in collaboration with the MEASURE DHS organization and the Bangladesh National Institute of Population Research and Training (NIPORT). BDHS is a representative probability sample of men and women based on a two-stage cluster sample of households, stratified by rural and urban areas and the seven administrative regions of the country. The primary sampling units (PSUs) in this survey corresponded with the most recent Census Enumeration Areas. On average each PSU contained 120 households. In the first stage of sampling, 600 PSUs were selected with probability proportional to the PSU size. In the second stage of sampling, 17,964 households were selected by systematic random sampling method. Of these, 17,511 were eligible. Interviews were successfully completed in 17,141 households. The overall response rate was 97.9%. In addition, one-third of the households were selected for biomarker information measurements including blood pressure and blood glucose assessment. In the biomarker sample, all men and woman aged 35 and older were eligible. In this subsample, 8,835 household members were available. Of these, 4,524 were men and 4,311 were women. After excluding non-respondents, final sample of 7,786 individuals was left for analysis. The overall response rate in the biomarker data was 89.2%. The sample selection framework is presented in Fig. 1. The detailed research protocol, methods, and structured questionnaires are available on the DHS website [29]. Blood pressure, blood glucose concentration, body weight, and height were assessed using standard methods, as previously described [29,31].

### Outcomes

The primary outcomes in our study were diabetes awareness, treatment, and control assessed through measurement and management of blood glucose. According to the ADA 2010 criteria, diabetes was defined as fasting blood glucose (FBG) values greater than or equal to 7.0 mmol/L or self-reported diabetes medication use. Awareness of diabetes was defined as answering ‘yes’ to the question ‘have you ever been told by a doctor or nurse that you had diabetes?’. Treatment was defined as current use of medication for diabetes. All participants were asked whether they received prescribed medications for their diabetes condition. Control of diabetes was defined as treated diabetes with FBG value less than 7.0 mmol/L.

### Covariates

We used individual, household, and community-level characteristics to assess the risk of awareness, treatment, and control of diabetes. The included individual level factors were respondent’s age (35–39, 40–44, 45–49, 50–54, 55–59, 60–69, ≥70 years), gender (man, women), marital status (currently married, not currently married), educational status (no education, primary, secondary, higher), current working status (yes, no), body mass index (BMI) (normal <25.0 kg/m^2^, overweight between 25.0 kg/m^2^ and <30.0 kg/m^2^, obese ≥30 kg/m^2^), and hypertension (yes, no). Respondents were classified as being hypertensive if their blood pressure values were systolic blood pressure (SBP) ≥140 mmHg or diastolic blood pressure (DBP) ≥90 mmHg, or if they reported currently taking antihypertensive medication. Household and community socio-economic conditions were classified into quintiles and tertiles, respectively. Respondent’s community type (rural, urban) and regional location (south, southeast, central, west, mid-western corner, northwest, and east) were treated as community level predictors.

### Statistical analyses

We used descriptive statistics and frequency distributions to describe participant characteristics. We estimated age-standardized prevalence of obesity, hypertension, and diabetes using Bangladesh Population Census 2001 data by direct standardized methods. To investigate the relationships between participant-, household-, and community-level characteristics and diabetes awareness we estimated two multilevel logistic regression model models with a random intercept at the household and community levels. The initial model included all selected characteristics and second or final model included only significant predictors (p<0.05) based on backward stepwise model-building. We used multilevel analysis because individuals are clustered within the same households and households are clustered within communities in BDHS data. Multilevel analysis produces more valid results when lower levels are nested within higher levels [32–34]. The major advantage of multilevel analysis is that it minimizes the effect of dependency between observations within sampling clusters [34]. We also computed a cubic spline regression model to evaluate the shape of the relationship of BMI with prevalence of diabetes, awareness, treatment, and control for diabetes. BMI equal to 25 kg/m^2^ was chosen as a reference value for estimating odds ratios and 95% CIs in cubic spline regression model. To adjust for missing data we used multiple imputation based on a regression model that estimates the missing value using known values to account for missing data [35,36]. Missing data were most frequent for observations of BMI (30.6%), followed by FBG (4.4%), SBP (0.2%), and DBP (0.2%). Similar to previous studies, age, sex, and place of residence were included as covariates in the imputation analysis [37]. All analyses at both the univariate and multilevel regression stages adjusted for the probability sample design. All statistical analyses were performed using Stata version 12.1/MP (StataCorp, College Station, Texas USA).

## Results

### Background characteristics

A total of 7,786 individuals aged 35 years or over participated in the study. Table 1 summarizes the crude and age-standardized characteristics of the study subjects. The mean age of the respondents was 51.4 years. The age-standardized mean BMI and FBG were 20.8 kg/m^2^ and 5.8 mmol/L, respectively. The overall age-standardized prevalence of prediabetes and diabetes was about 22% (95% CI 19.9–23.2) and 9% (95% CI 8.4–10.0), respectively. Among diabetic patients, about 41% (95% CI 36.4–45.9) were aware of their condition, 37% (95% CI 32.2–41.5) received treatment, and only 14% (95% CI 11.3–17.2) were able to control their blood glucose. Additionally, about 24.4% (95% CI 23.2–25.7) of the study subjects had hypertension. The detailed background characteristics of the participants’ economic condition by region of residence are presented in supplementary (S1 Table).

**Table 1:** Study population characteristics.

Characteristics	Crude	Age-standardized
**Mean (SE)**		
Age (years)	51.45 (0.17)	–
Weight (kg)	50.37 (0.14)	50.6 (0.13)
Height (mm)	1552.42 (0.97)	1553.43 (0.93)
Body mass index (kg/m^2^)	20.77 (0.05)	20.84 (0.04)
Systolic blood pressure (mmHg)	117.94 (0.34)	117.25 (0.33)
Diastolic blood pressure (mmHg)	77.52 (0.21)	77.43 (0.21)
Fasting blood glucose (mmol/L)	5.82 (0.03)	5.8 (0.03)
**Percentage (95% CI)**		
Obesity	0.9 (0.7–1.2)	0.9 (0.7–1.1)
Hypertension	25.4 (24.2–26.7)	24.4 (23.2–25.7)
Prediabetes	21.8 (20.2–23.5)	21.6 (19.9–23.2)
Diabetes	9.5 (8.7–10.3)	9.2 (8.4–10.0)
Awareness	42.7 (38.1–47.3)	41.2 (36.4–45.9)
Treatment	38.3 (34–42.8)	36.9 (32.2–41.5)
Control	15.0 (12.3–18.3)	14.2 (11.3–17.2)

SE, Standard error; 95% CI, 95% confidence interval

### Prevalence of diabetes and prediabetes

**Fig 2:**
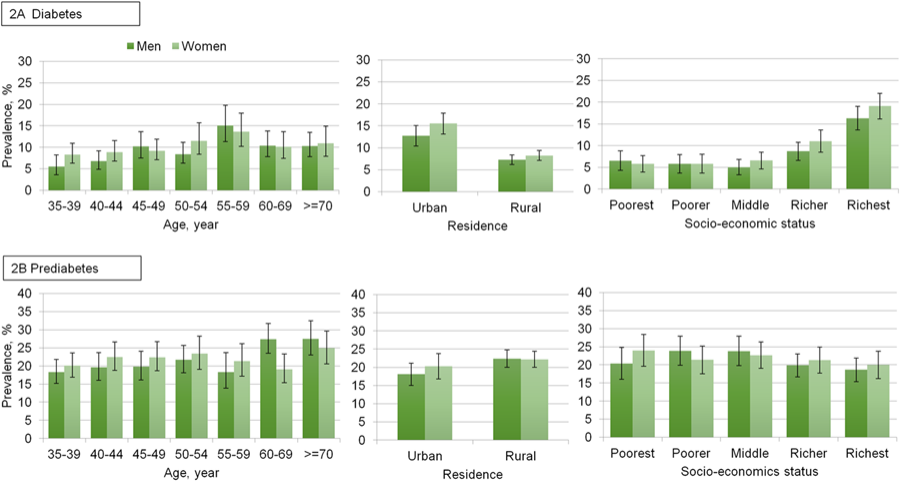
Age–specific and age–standardized prevalence of diabetes and prediabetes in Bangladeshi adults aged 35 years or older.

Age-standardized prevalence of prediabetes and diabetes according to sex with residence and household socio-economic status is presented in Fig. 2. Diabetes was more prevalent among aged 55–59 years in both sexes. The age-standardized prevalence of diabetes was higher in urban than rural residents. In addition, the prevalence of diabetes increased with the increase of socio-economic status, especially from middle class to richest quintile.

### Management of diabetes

The results of univariate analysis for diabetes awareness according to individual, household, and community characteristics are presented in Table 2. In addition, the age-standardized prevalence of awareness, treatment, and control of diabetes by different socio-demographic and health status are presented in supplementary (S2 Table). In univariate analyses (Table 2), diabetes awareness was significantly lower among the non-educated compared to the higher educated (25.8% vs. 67.8%), hypertensive persons versus non-hypertensive persons (55.3% vs. 34.2%), and poor residents versus wealthy residents (18.2% vs. 63.2%). In addition, residents who lived in the central part of Bangladesh had higher awareness (55.3%) of their diabetes, while lowest awareness (18.2%) was found in the northwestern region. Similar to diabetes awareness, participants who had no education and poor economic condition received treatment only 22.0%, and 15.8%, respectively (Table 2). Participants who lived in the central and eastern part of Bangladesh received diabetic treatment proportionally more than those in the northwestern region.

**Table 2:** Prevalence of awareness, treatment and control of diabetes.

Characteristics	Awareness(95% CI)	Treatment(95% CI)	Control(95% CI)
**Age group (years)**			
35–39	34.8 (25.1–45.8)	29.4 (20.4–40.2)	10.5 (5.7–18.5)
40–44	34.9 (25.5–45.6)	34.9 (25.5–45.7)	11.4 (6.2–20.3)
45–49	41.7 (32.0–52.1)	33.7 (24.8–43.9)	10.9 (5.9–19.1)
50–54	47.5 (37.2–58.1)	39.5 (29.1–50.8)	13.4 (7.5–22.8)
55–59	45.4 (34.8–56.6)	42.4 (31.9–53.6)	13.8 (6.7–26.3)
60–69	57.5 (46.0–68.2)	52.7 (41.4–63.8)	26.6 (18.1–37.4)
≥70	35.9 (27.1–45.9)	35.2 (26.4–45.1)	18.5 (12.2–27.0)
**Gender**			
Men	40.1 (34.1–46.3)	36.4 (30.6–42.6)	12.9 (9.2–17.8)
Women	45.0 (39.2–50.8)	39.9 (34.3–45.8)	16.9 (13.1–21.4)
**Educational status**			
No education	25.8 (20.2–32.2)	22.0 (16.9–28.2)	10.9 (7.3–15.9)
Primary education	40.6 (33.0–48.8)	37.0 (29.4–45.2)	13.9 (9.3–20.2)
Secondary education	55.0 (45.5–64.1)	48.8 (39.6–58.1)	16.7 (10.9–24.9)
Higher education	67.8 (58.5–75.9)	63.2 (53.9–71.6)	24.0 (16.2–34.1)
**Currently working**			
No	46.3 (40.7–52.0)	40.9 (35.6–46.5)	16.7 (13.2–20.8)
Yes	37.5 (31.0–44.5)	34.5 (28.1–41.4)	12.7 (8.5–18.5)
**Marital status**			
Currently married	42.6 (37.6–47.7)	37.7 (32.9–42.7)	13.9 (10.8–17.8)
Not currently married	43.1 (33.6–53.1)	41.0 (31.7–51.0)	20.0 (14.1–27.6)
**Hypertension**			
No	34.2 (28.9–40.0)	30.3 (25.1–36.0)	11.2 (8.2–15.1)
Yes	55.3 (48.3–62.0)	50.2 (43.5–57.0)	20.8 (15.4–27.4)
**Body mass index**			
Normal	38.9 (34.1–43.8)	34.7 (30.1–39.6)	13.4 (10.6–16.7)
Overweight	61.2 (50.7–70.7)	54.7 (45.7–63.4)	22.2 (14.3–32.8)
Obese	58.4 (33.7–79.5)	58.4 (33.7–79.5)	25.7 (9.0–54.6)
**Socio–economic status**			
Poorest	18.2 (10.4–29.8)	15.8 (8.8–26.8)	8.2 (3.7–17.1)
Poorer	12.1 (6.4–21.5)	10.1 (5.0–19.4)	10.1 (5.0–19.4)
Middle	31.0 (21.2–42.8)	25.8 (17.1–37.0)	11.2 (6.1–19.6)
Richer	42.6 (33.8–51.8)	40.2 (31.4–49.7)	17.6 (11.9–25.3)
Richest	63.2 (55.9–69.9)	56.6 (49.5–63.4)	18.4 (13.4–24.8)
**Place of residence**			
Urban	56.3 (48.6–63.8)	50.0 (42.4–57.6)	20.1 (14.7–26.8)
Rural	35.2 (29.9–40.8)	31.8 (26.9–37.2)	12.2 (9.4–15.7)
**Community status**			
Poor	17.4 (11.8–24.8)	16.7 (11.2–24.2)	10.2 (6.4–16.1)
Average	38.7 (30.8–47.2)	33.7 (26.0–42.4)	14.1 (9.6–20.2)
Rich	58.3 (51.6–64.7)	52.3 (46.0–58.6)	18.1 (13.7–23.5)
**Region of residence**			
Southern	24.2 (16.9–33.5)	21.2 (14.5–29.9)	10.1 (5.0–19.2)
Southeastern	42.8 (33.3–52.9)	37.6 (28.9–47.3)	8.0 (4.7–13.2)
Central	55.3 (45.1–65.2)	48.9 (39.3–58.7)	22.4 (16.2–30.1)
Western	37.1 (26.9–48.6)	33.5 (23.8–44.7)	7.7 (3.6–15.6)
Mid-western	40.4 (30.9–50.7)	35.7 (26.9–45.5)	16.8 (10.4–26.1)
Northwestern	18.2 (10.8–29.0)	19.1 (11.4–30.1)	8.6 (4.2–16.9)
Eastern	45.6 (33.3–58.5)	44.8 (32.1–58.2)	21.4 (14.3–30.6)

CI, 95% confidence interval

Table 3 shows multivariable analyses for risk factors of awareness of diabetes. The multilevel model for awareness of diabetes compared with a single level logistic regression model without random effects found a statistically significant difference (LR chi-squared (2) = 106.12, p<0.01 for initial model; LR chi-squared (2) = 106.79, p<0.0 1 for final model), which implies that random effect models are necessary to quantify the lack of diabetes awareness. In the multilevel final model, participants those who were older, primary or higher educated, had hypertension, belonged to the richest households, and those who lived in the central, mid-western, and eastern parts of Bangladesh were more likely to be aware of their diabetic condition.

**Table 3:** Risk factors of awareness of diabetes.

	Number of subjects		Odds ratio (95% CI)	
Characteristics	Not aware	Aware	Initial model	Final model
**Individual**				
Age group (years)				
35–39	73	37	1.00	1.00
40–44	77	38	0.84 (0.28–2.54)	0.83 (0.27–2.53)
45–49	79	52	1.41 (0.47–4.20)	1.38 (0.46–4.10)
50–54	52	54	3.81 (1.22–11.88)	3.53 (1.14–10.92)
55–59	56	47	1.85 (0.59–5.76)	1.75 (0.54–5.63)
60–69	56	60	5.73 (1.74–18.83)	4.64 (1.46–14.76)
≥70	65	50	3.62 (0.92–14.18)	2.95 (0.82–10.57)
Gender				
Men	227	152	1.00	-
Women	231	186	1.35 (0.53–3.45)	-
Educational status				
No education	196	66	1.00	1.00
Primary education	120	91	3.13 (1.30–7.53)	3.24 (1.37–7.68)
Secondary education	93	94	5.22 (1.88–14.46)	5.06 (1.91–13.44)
Higher education	49	87	12.40 (3.39–45.27)	12.03 (3.40–42.61)
Currently working				
No	243	217	1.00	1.00
Yes	215	121	0.40 (0.14–1.11)	0.32 (0.15–0.71)
Marital status				
Currently married	372	273	1.00	-
Not currently married	86	65	0.82 (0.31–2.13)	-
Hypertension				
No	317	157	1.00	1.00
Yes	141	181	2.82 (1.37–5.80)	2.75 (1.33–5.67)
Body mass index				
Normal	392	250	1.00	-
Overweight	57	77	1.17 (0.54–2.55)	-
Obese	9	11	0.32 (0.06–1.81)	-
**Household**				
Socio–economic status				
Poorest	78	13	1.00	1.00
Poorer	71	13	0.40 (0.08–2.03)	0.40 (0.08–2.08)
Middle	68	29	1.34 (0.30–5.93)	1.37 (0.30–6.12)
Richer	98	62	2.17 (0.53–8.81)	2.18 (0.54–8.84)
Richest	143	221	4.95 (0.99–24.71)	4.55 (0.94–22.04)
Community				
Place of residence				
Urban	174	191	1	-
Rural	284	147	1.78 (0.86–3.68)	-
**Community status**				
Poor	152	30	1.00	1.00
Average	121	68	3.20 (1.08–9.49)	3.09 (1.03–9.22)
Rich	185	240	3.97 (1.17–13.50)	3.06 (0.92–10.15)
Region of residence				
Southern	74	31	1.00	1.00
Southeastern	79	63	1.63 (0.53–5.03)	1.63 (0.53–4.98)
Central	60	80	6.33 (1.91–21.00)	5.78 (1.74–19.14)
Western	56	36	2.00 (0.57–6.95)	1.88 (0.53–6.64)
Mid-western	62	53	4.27 (1.32–13.83)	4.17 (1.29–13.40)
Northwestern	69	22	0.85 (0.23–3.17)	0.78 (0.21–2.91)
Eastern	58	53	3.30 (0.99–10.99)	3.40 (1.01–11.48)
Variance (cov.) of random effect				
Level 2 (Household)			3.89 (1.58)	4.13 (1.64)
Level 3 (Community)			1.22 (0.90)	1.20 (0.94)

Adjusted odds ratios (OR) with 95% confidence interval (CI) were reported from a multilevel logistic regression model accounting for intercept at household and community.

### Obesity and diabetes

**Fig 3:**
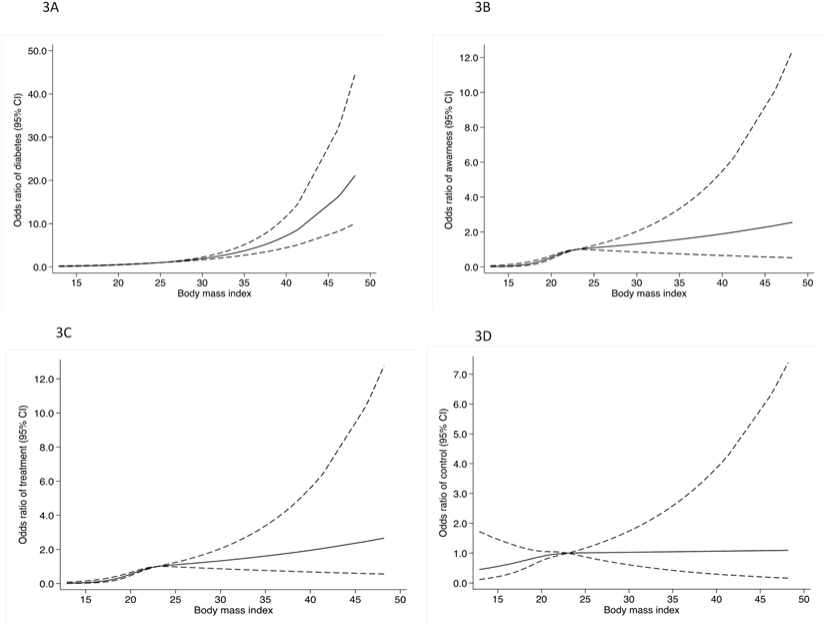
Cubic spline regression model between A) prevalence of diabetes and body much index (BMI), B) awareness of diabetes and BMI, C) treatment of diabetes and BMI, and D) control of diabetes and BMI. Spline regression (three knots, reference value: BMI = 25 kg/m^2^). Continuous line, odds ratio for diabetes and dashed line, 95% confidence interval. The model was adjusted for age and sex.

Fig. 3 shows spline regression model for the association between A) BMI and prevalence of diabetes, B) BMI and awareness of diabetes, C) BMI and treatment of diabetes D) BMI and control of diabetes. There was a threshold effect on the association between BMI and diabetes. We observed a plateau for prevalence of diabetes associated with BMI< 30 kg/m^2^, while for BMI≥ 30 kg/m^2^ prevalence of diabetes increased sharply with increasing BMI. A non-linear relationship was also observed for the association of BMI with awareness, treatment and control for diabetes.

## Discussion

Non-communicable diseases especially diabetes mellitus have become a major health concern in low-income countries. Our study is one of the few studies that addressed awareness, treatment, and control of diabetes in Bangladeshi adults using nationwide population-based survey data. We found an overall high prevalence of diabetes and low prevalence of awareness, treatment, and control. A wide gap was found in our study between detection and control of diabetes across non-educated to higher educated and poor to rich households. Our study findings revealed that participants of younger age, in poverty, with no education, and residence in the northwestern part of Bangladesh were less likely to receive treatment for diabetes and control their condition.

The present study found that about one in ten Bangladeshi adults had diabetes (9.2%). This finding is consistent with many other studies from low- and middle-income countries including India (8.6%) [38], Sri Lanka (8.0%) [39], China (9.6%) [40], Nauru (13.7%) [41], and Panama (9.5%) [12]. According to the 2013 estimates by the International Diabetes Federation (IDF), the age-standardized prevalence of diabetes was 6.3% in Bangladesh, which was lower than our present estimate. Comparison of prevalence within and between countries are difficult because of several differences in methods including: different regional focus, inadequate sample size, varied sample design, limited focus on both rural and urban population, different age distribution, and lack of uniform diagnostic criteria. We estimated prevalence of diabetes using 2011 Bangladesh DHS data, which was the first nationally representative study in Bangladesh. Recently, a meta-analysis found an increasing trend in the prevalence of diabetes among Bangladeshi adults [42]. Consistent with other studies from Asia [17,43], prevalence of diabetes in our study increased disproportionately among the young and middle-aged and rich households. Respondents who were women, belonged to rich households and lived in urban areas had a relatively higher prevalence of diabetes. This could be due to lack of physical activity, sedentary lifestyle, and unhealthy dietary habits of rich women who lived in urban areas.

In our study, about 41.2% of diabetic subjects were aware of their condition, 36.9% received treatment, and only 14.2% controlled their condition. Similar prevalence of diabetes awareness was also reported in China (45.8%) [6] and India (36.0%) [44]. According to the International Diabetes Federation, around half of South Asian people with diabetes were unaware of their condition in 2013 [1]. We cannot justify our findings regarding management of diabetes in Bangladesh due to absence of literature. Compared to our findings, slightly higher prevalence of diabetes control was observed in other countries including India (16.9%) [44] Thailand (21.6%) [15] and China (20.9%) [6]. This difference may be due to country specific disease management programs and health system performance. Our study findings indicated that the diagnosis, treatment, and control rates of diabetes in Bangladesh significantly differed by education level, household socio-economic status, and region of residence. Similar to other studies from developing and developed countries [13,18,45], a striking variation of diabetes treatment was found by education (22% non-educated versus 63% higher educated) and socio-economic status (16% poor versus 57% richest households). In addition, participants who lived in the poorer region (northwestern part of Bangladesh) were less likely to be aware of their diabetes condition. Awareness and treatment were also quite low among subjects with no education, lower socio-economic status, and those who lived in rural areas and poor communities. Overall, diabetes management in the general Bangladeshi population remains disproportionately and substantially low, which could raise concern for higher rates of death, disability, and household economic shock in the near future.

Our study together with a previous study confirms that prevalence of diabetes is increasing in Bangladesh [42]; however treatment and control are substantially low. There are several reasons for this variation of awareness and treatment of diabetes among populations in low-income countries like Bangladesh. Access to care is closely related to household economic status, which could affect awareness and treatment. A previous study from Bangladesh suggested that the poor population had less capacity to spend on healthcare [1]. Secondly, awareness and treatment of diabetes highly rely on the ability of the health system to provide diagnosis and other services with affordable care to the general population. A recent study from Bangladesh suggested that more than 12% of households borrowed money or sold household assets to pay health care costs related to chronic diseases [11]. Although public health services are subsidized by the government [46], they are also unable to provide affordable care for the poor population [10,11]. This implies that subsidized programs may not be working properly among this subpopulation. Therefore, the poor population in Bangladesh may skip taking medicine or refrain from accessing health services to avoid financial burden associated with treatment costs. Despite the epidemiological transitions and financial burden, the health care system in Bangladesh is highly restricted to communicable diseases especially maternal and child health programs [47]. NCD management especially diabetes and hypertension receive less attention. The health policies and programs in Bangladesh should be scaled up according to the current and predicted disease burden.

The present study has several strengths. The main strengths are the nationally representative population-based survey and the coverage of both rural and urban areas. The findings provided detailed information on a wide range of risk factors for awareness of diabetes among the adult population in Bangladesh while considering probability weights, and clustering effects. However, the present study has several limitations. First, it was unable to identify causal effects, as the study is cross-sectional in nature. Second, it was not able to assess the association between outcome variables and some important variables like duration of diabetes mellitus, dietary intake, smoking status, lifestyle behaviors, and level of physical exercise. Moreover, the present study was unable to control for or assess the independent effects of these factors on the prevalence of awareness, treatment, and control of diabetes mellitus. Third, due to small sample size for variables treatments and controls of diabetic among individuals those who were aware of their conditions, it was not possible to assess the risk factors of treatment and control using multilevel logistic regression analysis.

In conclusion, this population-based cross-sectional study suggests that people in higher socio-economic status and those living in urban areas have higher rates of diabetes. Among diabetics 41.2% were aware of the diagnosis, 36.9% were treated, and only 14.2% controlled their blood glucose level. People with no education, lower socio-economic status, and those who lived in disadvantaged regions in terms of education and economic profile (northwestern part of Bangladesh) were found lacking of diagnosis, treatment, and control of diabetes. The findings from our study suggest that substantial improvements of diabetes detection and treatment are needed in Bangladesh especially among disadvantaged populations. These can be tackled by (i) reforming the health system based on disease burden, the Government of Bangladesh should give top priority to NCDs especially diabetes prevention and control in their health promotion programs; (ii) implementing universal health insurance or other risk pooling mechanisms in health financing system to ensure access and affordable care for all citizen from poor to rich; and (iii) creating diabetes awareness, changing lifestyle and dietary habits through well-designed public education and mass media campaigns.

## Supporting Information

S1 Table Household and community socio-economic status by location of residence. (DOC)

S2 Table Age-standardized prevalence of awareness, treatment and control of diabetes (N = 796). (DOC)
