# Supplementary material for: Awareness, Treatment, and Control of Diabetes in Bangladesh: A Nationwide Population-Based Study
Source: PLoS One. 2015 Feb 18;10(2):e0118365. doi: 10.1371/journal.pone.0118365 (PMC4334658; doi:10.1371/journal.pone.0118365)
Supplement: S1 Table — (DOC) [file pone.0118365.s001.doc]

**Table S1 Household and community socio-economic status by location of residence**

| **Characteristics** | **Percentage of population according to location of residence** | | | | | | |
| --- | --- | --- | --- | --- | --- | --- | --- |
|  | **Southern** | **Southeastern** | **Central** | **Western** | **Mid- western** | **Northwestern** | **Eastern** |
| **Malnutrition** | 26.1 | 21.1 | 20.4 | 19.0 | 19.4 | 26.9 | 24.8 |
| **Household socio-economic status** |  |  |  |  |  |  |  |
| Poorest | 17.8 | 13.7 | 20.3 | 16.7 | 19.6 | 27.4 | 22.7 |
| Poorer | 26.3 | 19.0 | 14.7 | 16.1 | 23.0 | 27.9 | 18.0 |
| Middle | 29.1 | 21.0 | 15.8 | 23.8 | 22.8 | 17.2 | 17.5 |
| Richer | 17.6 | 25.3 | 19.7 | 22.2 | 19.9 | 18.8 | 17.2 |
| Richest | 9.2 | 21.0 | 29.4 | 21.2 | 14.7 | 8.7 | 24.7 |
| **Community economic status** |  |  |  |  |  |  |  |
| Poor | 54.2 | 23.3 | 32.8 | 14.5 | 43.4 | 51.5 | 35.6 |
| Average | 31.4 | 41.3 | 25.6 | 50.6 | 32.4 | 37.2 | 21.9 |
| Rich | 14.3 | 35.4 | 41.5 | 34.9 | 24.3 | 11.3 | 42.6 |
