# Supplementary material for: Awareness, Treatment, and Control of Diabetes in Bangladesh: A Nationwide Population-Based Study
Source: PLoS One. 2015 Feb 18;10(2):e0118365. doi: 10.1371/journal.pone.0118365 (PMC4334658; doi:10.1371/journal.pone.0118365)
Supplement: S2 Table — (DOC) [file pone.0118365.s002.doc]

**Table S2** Age-standardized prevalence of awareness, treatment and control of diabetes (N=796)

| **Characteristics** | **Age-standardized prevalence (95% CI)** | | |
| --- | --- | --- | --- |
|  | **Awareness** | **Treatment** | **Control** |
| **Gender** |  |  |  |
| Men | 35.6 (29.5–41.6) | 32.7 (26.6–38.8) | 11.5 (7.40–15.6) |
| Women | 44.5 (38.6–50.5) | 39.1 (33.3–45.0) | 16.3 (12.3–20.3) |
| **Educational status** |  |  |  |
| No education | 25.2 (18.3–32.1) | 20.0 (13.8–26.1) | 9.1 (4.8–13.3) |
| Primary education | 38.3 (30.6–46.0) | 35.1 (27.5–42.7) | 13.0 (8.0–17.9) |
| Secondary education | 54.5 (45.4–63.6) | 49.3 (40.2–58.4) | 17.2 (10.6–23.8) |
| Higher education | 62.9 (53.9–72.0) | 59.2 (49.9–68.5) | 23.4 (14.9–31.8) |
| **Currently working** |  |  |  |
| No | 45.4 (39.3–51.6) | 39.5 (33.6–45.5) | 15.1 (11.2–19.0) |
| Yes | 35.0 (28.3–41.8) | 32.5 (25.8–39.2) | 12.7 (7.8–17.6) |
| **Marital status** |  |  |  |
| Currently married | 41.3 (36.2–46.4) | 36.6 (31.7–41.6) | 13.8 (10.5–17.1) |
| Not currently married | 31.8 (19.5–44.0) | 30.4 (18.1–42.7) | 12.2 (7.6–16.8) |
| **Hypertension** |  |  |  |
| No | 33.7 (28.1–39.3) | 29.9 (24.4–35.4) | 11.4 (8.0–14.8) |
| Yes | 53.2 (45.0–61.5) | 46.9 (38.9–54.9) | 18.7 (12.3–25.1) |
| **Body mass index** |  |  |  |
| Normal | 38.3 (33.3–43.3) | 34.1 (29.2–39.1) | 13.0 (10.1–15.9) |
| Overweight | 53.9 (40.9–66.9) | 49.4 (36.4–62.4) | 18.8 (8.1–29.5) |
| Obese | 69.7 (57.1–82.2) | 69.7 (57.1–82.2) | 15.6 (4.2–27.0) |
| **Socio–economic status** |  |  |  |
| Poorest | 17.7 (7.8–27.5) | 14.6 (5.70–23.5) | 6.8 (1.7–11.9) |
| Poorer | 14.5 (4.4–24.7) | 9.90 (2.70–17.1) | 9.9 (2.7–17.1) |
| Middle | 27.3 (17.2–37.3) | 22.9 (13.4–32.4) | 8.6 (3.7–13.5) |
| Richer | 42.5 (33.4–51.5) | 39.3 (30.3–48.3) | 16.4 (9.9–22.8) |
| Richest | 58.0 (51.0–65.0) | 52.8 (45.6–59.9) | 17.6 (12.2–23.0) |
| **Place of residence** |  |  |  |
| Urban | 54.0 (45.9–62.2) | 47.5 (39.6–55.4) | 19.9 (13.9–25.9) |
| Rural | 33.9 (28.3–39.5) | 30.8 (25.3–36.2) | 11.2 (8.3–14.2) |
| **Community status** |  |  |  |
| Poor | 15.6 (9.1–22.2) | 14.6 (8.3–21.0) | 8.2 (4.2–12.2) |
| Average | 38.6 (30.0–47.1) | 33.6 (25.0–42.3) | 13.1 (8.3–17.8) |
| Rich | 56.0 (49.3–62.8) | 50.5 (43.9–57.2) | 17.9 (13–22.7) |
| **Region of residence** |  |  |  |
| Southern | 21.7 (13.7–29.8) | 18.3 (11.0–25.6) | 8.3 (2.6–14.0) |
| Southeastern | 43.5 (33.8–53.1) | 38.6 (29.0–48.2) | 8.9 (4.7–13.2) |
| Central | 52.5 (42.4–62.7) | 46.4 (36.4–56.4) | 20.2 (13.7–26.8) |
| Western | 36.6 (24.2–49.0) | 34.0 (21.4–46.6) | 5.5 (2.1–8.9) |
| Mid-western | 37.0 (28.3–45.6) | 32.5 (24.7–40.3) | 15.9 (9.2–22.6) |
| Northwestern | 15.9 (8.2–23.5) | 16.2 (8.20–24.2) | 8.4 (2.3–14.5) |
| Eastern | 42.7 (29.6–55.8) | 43.1 (30.1–56.1) | 20.0 (11.9–28.0) |
